# Management of internal iliac and obturator lymph nodes in mid-low rectal cancer

**DOI:** 10.1186/s12957-024-03427-0

**Published:** 2024-06-12

**Authors:** Tixian Xiao, Jianan Chen, Qian Liu

**Affiliations:** 1 Department of Colorectal Surgery, National Cancer Center/National Clinical Research Center for Cancer/Cancer Hospital, Chinese Academy of Medical Sciences and Peking Union Medical College, Beijing, 100021 China; 2 Fred & Pamela Buffett Cancer Center, University of Nebraska Medical Center, 68198 Omaha, Nebraska USA

**Keywords:** Rectal cancer, Lateral pelvic lymph nodes, Internal iliac lymph nodes, Obturator lymph nodes

## Abstract

In rectal cancer treatment, the diagnosis and management of lateral pelvic lymph nodes (LLN) are critical for preventing local recurrence. Over time, scholars have reached a consensus: when imaging suggests LLN metastasis, combining neoadjuvant chemoradiotherapy (nCRT) with selective LLN dissection (LLND) can mitigate the risk of recurrence. Selective LLND typically encompasses lymph nodes in the internal iliac and obturator regions. Recent studies emphasize distinctions between internal iliac and obturator lymph nodes regarding prognosis and treatment outcomes, prompting the need for differentiated diagnostic and treatment approaches.

## Treatment of lateral pelvic lymph nodes

Positive lateral pelvic lymph nodes (LLN) significantly contribute to local recurrence in rectal cancer patients [1]. There are differing opinions regarding the treatment of LLN. Japanese scholars endorse a combination of total mesorectal excision (TME) and lateral lymph node dissection (LLND) for LLN, whereas Western countries favor a combination of neoadjuvant chemoradiotherapy (nCRT) and TME. Accumulating research supports a comprehensive treatment approach combining nCRT with selective LLND when suspicious lateral lymph node metastasis is identified through imaging, effectively reducing the risk of local recurrence [2–4].

Nevertheless, disagreement persists regarding the extent of LLND clearance. Chinese scholars recently reached a consensus that lymph node metastasis in the internal iliac and obturator fossa regions should be classified as regional lymph node metastasis and included in the clearance range. In contrast, the external iliac and common iliac regions are categorized as metastatic disease, and performing LLND in these areas does not yield survival benefits [5]. According to the American Joint Committee on Cancer (AJCC) Cancer Staging Manual, Eighth Edition, internal iliac lymph nodes in rectal cancer are defined as regional lymph nodes, whereas obturator, external iliac, and common iliac lymph nodes are classified as metastatic disease [6]. Akiyoshi [7] analyzed a nationwide multi-institutional database in Japan and discovered that patients with affected internal iliac lymph nodes exhibited outcomes comparable to those with N2a disease and fared better than those with N2b disease. Interestingly, patients with lateral lymph node metastasis beyond the internal iliac region demonstrated better survival rates than stage IV patients who underwent curative resection. Consequently, this research advocates for classifying all lateral lymph node metastases as regional disease.

## Definition of internal iliac and obturator regions

Precisely defining the boundaries of the internal iliac and obturator regions is essential for accurate neoadjuvant radiotherapy and surgical planning. However, variations exist in how radiation oncologists and surgeons interpret these regions. Presently, the Dutch Radiotherapy Delineation Guideline (LPRGE consensus 2023) has been refined to mitigate confusion within multidisciplinary teams (MDTs) [8].

From a surgical anatomical perspective, internal iliac lymph nodes are situated around the internal iliac artery and its branches, including the superior vesical artery, inferior vesical artery (or vaginal artery), umbilical artery, and obturator artery. These nodes extend between the left and right inferior hypogastric nerves, downward to the pelvic plexus, and posteriorly to the Alcock canal. Further division of internal iliac nodes includes the distal internal iliac vessels (No. 263d) and the proximal internal iliac vessels (No. 263p), separated by the superior vesical artery. The inner boundary extends from the ureter to the fascia covering the pelvic plexus, while the outer boundary aligns with the lateral edges of various internal iliac vessel branches. This outer boundary serves as the demarcation between the internal iliac and obturator lymph node regions.

**Fig. 1:**
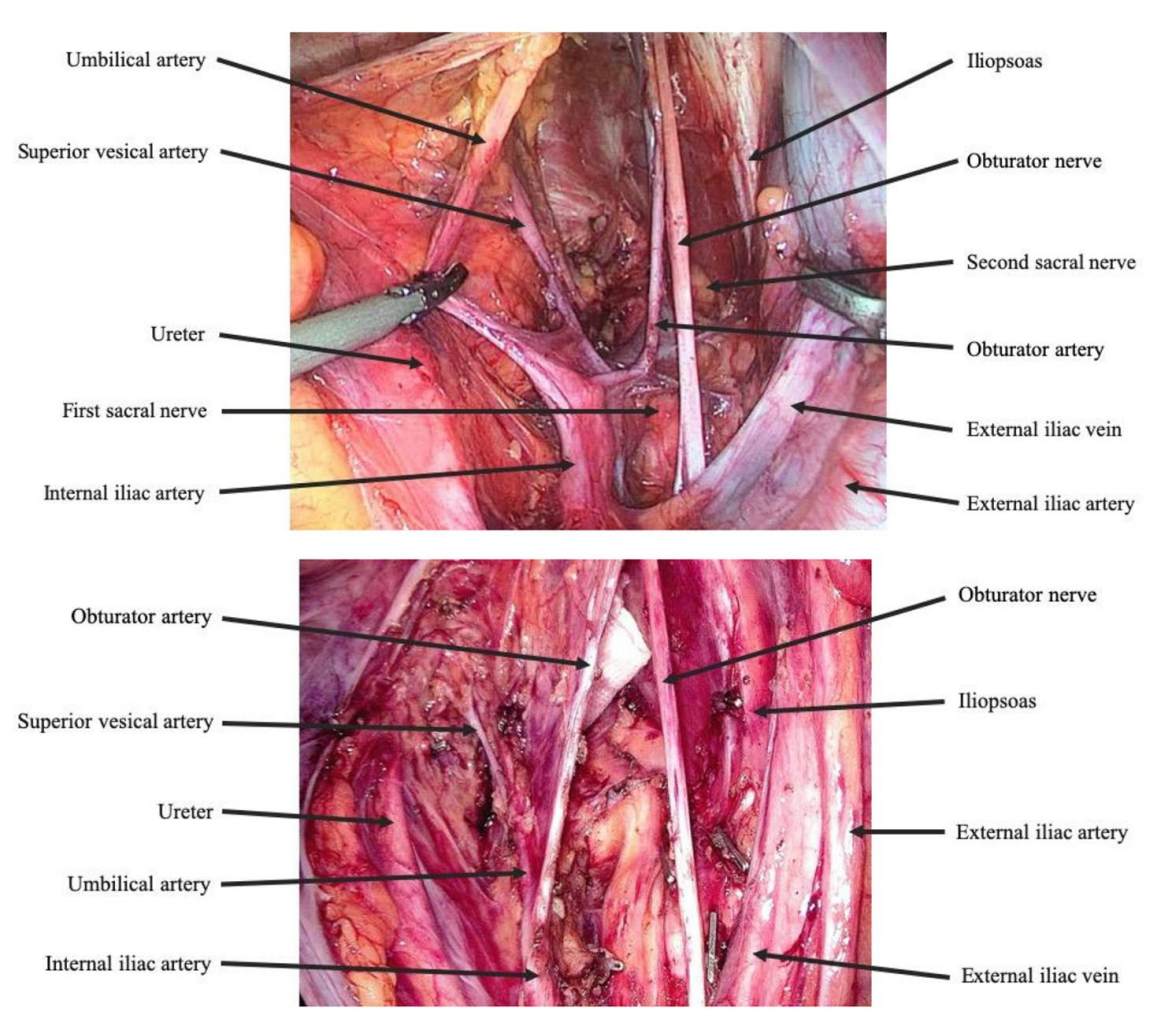
The important anatomical structures and locations of lymph nodes in the internal iliac and obturator regions are crucial

Obturator lymph nodes (No. 283) are located along the obturator artery. Their inner extension reaches the internal iliac artery and superior vesical artery (outer surface of the fascia covering the bladder). The outer extension encompasses the obturator artery and pelvic wall (along the inner edge of the psoas major muscle and obturator internus muscle). Posteriorly, they extend toward the sciatic nerve and piriformis muscle [9–14] (Fig. 1).

## Imaging diagnosis

Presently, magnetic resonance imaging (MRI) of the rectum stands as the most accurate preoperative diagnostic method for assessing lateral pelvic lymph node (LLN) positivity [15, 16]. MRI images enable measurement of the diameter of enlarged LLN. A multicenter study by the International Rectal Cancer Lateral Lymph Node Assistance Group suggests a short-axis diameter of 7 mm as the optimal cutoff value for pre-nCRT LLN assessment to determine lateral lymph node metastasis [4, 17]. Nevertheless, no research currently reports the optimal short-axis diameter cutoff value for assessing internal iliac or obturator lymph node metastasis before nCRT.

MRI images also can identify malignant features in lymph nodes, including internal heterogeneity and irregular borders, which serve as predictors of metastatic potential [18, 19]. Notably, even moderately sized LLN (5.0–6.9 mm) with at least one malignant feature carry an 8% risk of local recurrence within 4 years [20].

Recent retrospective studies from the Netherlands reveal that nearly half of the initial MRI reports for rectal cancer patients lack relevant information about LLN. Furthermore, even when lateral lymph node enlargement is reported, incomplete or contradictory descriptions of lymph node features occur. These findings underscore the need for enhanced awareness, improved reporting templates, and targeted education and training regarding LLN [21–23].

Moreover, researchers have recently employed radiomics-based methods on MRI images to predict lateral pelvic lymph node metastasis in locally advanced rectal cancer. These radiomics approaches outperform simple short-axis diameter measurements. Specifically, compared to pre-treatment short-axis diameter measurements, radiomics scores demonstrated significantly better discrimination in both the primary cohort (area under the curve, AUC 0.91 vs. 0.83, *p* = 0.0015) and validation cohort (AUC 0.90 vs. 0.80, *p* = 0.0298) [24]. When compared to clinical models (AUC = 0.772; 95% CI, 0.589–0.856) and radiomics models (AUC = 0.731; 95% CI, 0.613–0.849), the combined clinical-radiomics model exhibited the highest discriminatory ability (AUC = 0.843; 95% CI, 0.706–0.968) [25].

Furthermore, computed tomography (CT) and positron emission tomography/ computed tomography (PET/CT) offer diagnostic potential. Post-nCRT, PET/CT—either alone or in conjunction with CT and MRI—accurately predicts the presence of metastatic LLN. Specifically, when the size is ≥ 12 mm and/or the standardized uptake value (SUV) is ≥ 1.6, high accuracy, sensitivity, and specificity are achieved [26]. Integrating CT texture analysis with conventional diagnostic imaging may further enhance the accuracy of diagnosing LLN metastasis in rectal cancer [27].

## Neoadjuvant chemoradiotherapy

In the treatment process of LLND, a consensus has emerged to prioritize nCRT [28–30]. The combination of nCRT and LLND effectively controls recurrence in the obturator area (0.4%). However, recurrence rates are higher in the internal iliac and pelvic nerve regions (6.6%) [31, 32]. Patients with internal iliac and obturator lymph node metastases exhibit response rates of approximately 22% and 63%, respectively. Notably, patients with internal iliac lymph node metastases who do not respond to neoadjuvant treatment experience an increased 5-year lateral local recurrence (LLR) rate. Similarly, patients with no treatment response and obturator lymph node metastases demonstrate a slightly elevated 5-year LLR rate compared to those with minimal or responsive disease, although this increase lacks statistical significance [33].

Following neoadjuvant (chemotherapy) radiotherapy, the obturator lymph node metastasis (distal lateral compartment) group exhibits significantly improved disease-free survival (DFS) compared to the internal iliac lymph node metastasis (proximal lateral compartment) group (hazard ratio [HR] = 0.370; 95% confidence interval [CI] = 0.144–0.846; *p* = 0.020). Furthermore, the overall survival (OS) trend in the obturator lymph node metastasis (distal lateral compartment) group surpasses that in the internal iliac lymph node metastasis (proximal lateral compartment) group (HR = 0.308; 95% CI = 0.055–1.275; *p* = 0.120) [34].

Despite the overall risk reduction in local recurrence due to nCRT, certain patients with internal iliac or obturator lymph node metastases may not attain adequate local control [29, 31]. Remarkably, even after nCRT, clinically suspicious pelvic sidewall lymph nodes persistently harbor pathologically confirmed metastases in 25–85% of cases [35, 36]. Notably, patients with lymph nodes measuring > 4∼6 mm on MRI restaging continue to exhibit elevated rates of local recurrence or positive lymph node pathology (ranging from 40.3 to 75.0%) [37].

## Surgical treatment for lymph node metastasis

Despite nCRT, patients with internal iliac and obturator lymph node metastases may still harbor suspicious lymph node metastases [38–40]. Selective LLND offers survival benefits, given that over 95% of lateral lymph node metastases occur within the internal iliac vessels and obturator area [41, 42]. Short diameters of internal iliac lymph nodes (≥ 4 mm) and obturator lymph nodes (≥ 6 mm) after nCRT serve as risk factors for lateral recurrence [33, 43].

Achieving a balance between thorough lymph node clearance and preservation of vascular and neural structures within the surgical area remains a critical consideration for surgeons. Internal iliac lymph nodes predominantly cluster around the bladder arteries (or vaginal arteries, with the rectal artery often sharing a common origin). Notably, approximately 44% of positive lymph nodes are distributed in the more distant interval below the piriformis muscle, while fewer occur around the uterine and superior vesical arteries. Given the intricate and variable branching patterns of internal iliac vessels, meticulous preoperative assessment of the relationship between metastatic lymph nodes and adjacent vessels and nerves is essential [34, 44]. Research findings suggest that ligating internal iliac vessels does not result in pelvic organ ischemia in the vast majority of cases [11, 44, 45].

Findings from the JCOG0212 study indicate that preserving autonomous nerves during LLND does not increase the incidence of postoperative urinary dysfunction compared to conventional TME [46]. Immunohistochemical studies of resected specimens reveal that the pelvic autonomous nerve plane lacks lymph node tissue, providing theoretical support for nerve preservation [47]. Performing LLND for rectal cancer guided by fascial planes, rather than a vessel-oriented approach, better safeguards the pelvic autonomous nerves and reduces the risk of postoperative urinary and male reproductive dysfunction [10, 48].

The advent of robotic surgery enables surgeons to enhance their visualization of neural and vascular structures during LLND, facilitated by the robot arms that offer greater surgical flexibility. Existing research supports the feasibility of this approach [49–52].

Visualizing LLN is crucial for precise surgical dissection. Consequently, some surgeons explore the integration of 3D printing technology {Hojo, 2019 #75}. In a single-center study from Japan, 3D printing technology was employed to create a pelvic model for surgical reference, leading to shorter operative times (median time 458 min vs. 558 min) and an increased lymph node yield (median 9 nodes vs. 6 nodes) [53].

Identifying the precise location of LLN takes precedence during surgery. Indocyanine green (ICG) fluorescence imaging has shown favorable outcomes in LLND based on several studies. Compared to non-ICG groups, the ICG group exhibited significantly reduced intraoperative blood loss (55.8 ± 37.5 mL vs. 108.0 ± 52.7 mL, *p* = 0.003) and increased harvested lateral lymph node numbers (11.5 ± 5.9 vs. 7.1 ± 4.8, *p* = 0.017) [54]. Remarkably, the 3-year cumulative lateral local recurrence rate was 0% in the ICG group compared to 9.3% in the non-ICG group (*p* = 0.048) [55]. However, a notable limitation lies in the technique’s reliance on the penetration depth of laser light to excite the fluorescent dye, rendering it visible to the camera system. In most cases, the penetration depth is only 1–2 mm, limiting its applicability in the distant end of internal iliac vessel lymph node dissection [3].

In addition, some researchers have explored new surgical approaches. Transanal-assisted LLND emerges as a potentially superior surgical approach. In comparison to the traditional transabdominal approach, transanal-assisted procedures result in shorter operative times and reduced blood loss [56, 57]. However, ongoing debate surrounds whether this approach correlates with lower postoperative complication rates and increased lateral lymph node yield. Notably, there is currently no relevant research on the long-term prognosis of patients undergoing transanal-assisted procedures.

## Postoperative adjuvant treatment and prognosis

In cases where patients have not undergone preoperative chemoradiotherapy and exhibit positive lymph node pathology after surgery, the local recurrence rate can range from 22.2 to 56.8% [58, 59]. Routine addition of adjuvant radiotherapy following surgery is recommended. However, current evidence from well-designed studies remains insufficient to definitively establish whether additional adjuvant chemotherapy after nCRT confers survival benefits [60].

Following radiotherapy and chemotherapy, the recurrence period for LLN may extend beyond the immediate postoperative phase. International multicenter studies reveal that a significant proportion of internal iliac lymph node recurrences occur 3 years or more after neoadjuvant treatment. Specifically, patients with internal iliac lymph nodes measuring greater than 4 mm in diameter experience 3-year and 5-year recurrence rates of 37.0% and 52.3%, respectively. Conversely, patients with lymph nodes measuring 4 mm or less exhibit 3-year and 5-year recurrence rates of 0% and 20.0%, respectively. Similarly, for obturator lymph nodes measuring greater than 6 mm, the 3-year and 5-year recurrence rates are 17.8%, whereas for those measuring 6 mm or less, the rates remain at 0% [43].

Typically, internal iliac lymph node metastasis is categorized as regional lymph node involvement and exhibits a prognosis comparable to N2a patients. However, patients with obturator lymph node metastasis experience a worse prognosis than those with internal iliac lymph node metastasis [7, 33].

The 4-year lateral local recurrence rate for internal iliac lymph nodes stands at approximately 7%, whereas for obturator lymph nodes, it exceeds 17% [61]. A retrospective study utilizing data from the Japanese Society for Cancer of the Colon and Rectum (JSCCR) reported that OS and relapse-free survival (RFS) of patients with obturator lymph node metastasis were slightly inferior to those with internal iliac lymph node metastasis (OS: HR 3.88, 95% CI 2.89–5.21; RFS: HR 3.15, 95% CI 2.43–4.09), although these differences did not reach statistical significance (*p* = 0.095 and 0.075) [62].

## Looking to the future

At present, internal iliac and obturator lymph nodes share similar diagnostic and treatment approaches. Nevertheless, as research advances, notable distinctions emerge between internal iliac lymph nodes and obturator lymph nodes concerning diagnostic criteria, treatment response, and overall patient prognosis. Consequently, it becomes essential to differentiate their diagnostic and treatment strategies. Yet, existing studies primarily rely on retrospective designs, often featuring limited sample sizes and substantial selection biases. Consequently, they do not yield high-level research evidence for precisely treating these two diseases. The ongoing LaNoReC study, an international prospective registry focusing on patients with rectal cancer and LLN metastases in the internal iliac and/or obturator region, is currently enrolling participants. This study holds promise for supplying additional data that can guide future clinical decision-making.

## Data Availability

No datasets were generated or analysed during the current study.
